# Resistin as a potential diagnostic biomarker for sepsis: insights from DIA and ELISA analyses

**DOI:** 10.1186/s12014-024-09498-1

**Published:** 2024-07-01

**Authors:** Youyu Lan, Wentao Guo, Wenhao Chen, Muhu Chen, Shaolan Li

**Affiliations:** 1 Department of Rheumatology and Immunology, the Affiliated Hospital of Southwest Medical University, 25 Taiping Street, Jiangyang District, Luzhou, 646000 Sichuan China; 2 Department of Emergency Medicine, The Affiliated Hospital, Southwest Medical University, Jiangyang District, Luzhou City, 646000 Sichuan Province China

**Keywords:** Bioinformatics, Sepsis, Resistin, DIA, ELISA

## Abstract

**Purpose:**

The primary objective of this investigation is to systematically screen and identify differentially expressed proteins (DEPs) within the plasma of individuals afflicted with sepsis. This endeavor employs both Data-Independent Acquisition (DIA) and enzyme-linked immunosorbent assay (ELISA) methodologies. The overarching goal is to furnish accessible and precise serum biomarkers conducive to the diagnostic discernment of sepsis.

**Method:**

The study encompasses 53 sepsis patients admitted to the Affiliated Hospital of Southwest Medical University between January 2019 and December 2020, alongside a control cohort consisting of 16 individuals devoid of sepsis pathology. Subsequently, a subset comprising 10 randomly selected subjects from the control group and 22 from the sepsis group undergoes quantitative proteomic analysis via DIA. The acquired data undergoes Gene Ontology (GO) and Kyoto Encyclopedia of Genes (KEGG) analyses, facilitating the construction of a Protein-Protein Interaction (PPI) network to discern potential markers. Validation of core proteins is then accomplished through ELISA. Comparative analysis between the normal and sepsis groups ensues, characterized by Receiver Operating Characteristic (ROC) curve construction to evaluate diagnostic efficacy.

**Result:**

A total of 187 DEPs were identified through bioinformatic methodologies. Examination reveals their predominant involvement in biological processes such as wound healing, coagulation, and blood coagulation. Functional pathway analysis further elucidates their engagement in the complement pathway and malaria. Resistin emerges as a candidate plasma biomarker, subsequently validated through ELISA. Notably, the protein exhibits significantly elevated levels in the serum of sepsis patients compared to the normal control group. ROC curve analysis underscores the robust diagnostic capacity of these biomarkers for sepsis.

**Conclusion:**

Data-Independent Acquisition (DIA) and Enzyme-Linked Immunosorbent Assay (ELISA) show increased Resistin levels in sepsis patients, suggesting diagnostic potential, warranting further research.

## Introduction

Sepsis, a prevalent syndrome characterized by acute and heightened mortality and disability rates, is delineated as multiple organ dysfunction arising from immune dysfunction triggered by infection. In the United States, the annual incidence of infections may escalate to 1.7 million, resulting in over 250,000 fatalities. Survivors contend with diverse chronic complications, markedly diminishing their quality of life. Presently, treatment modalities predominantly adhere to empirical and symptomatic approaches, encompassing broad-spectrum antimicrobial agents, fluid resuscitation, anti-shock measures, infection prevention, vasoactive drugs, among others. Targeted therapeutic interventions addressing etiology and immune response are limited [1]. The economic burden of sepsis treatment ranks among the highest within hospitalized diseases. Initiated in 2002, the Sepsis Survivor Movement promulgated evidence-based treatments aiming to curtail associated mortality rates. However, the complexity, heterogeneity, and formidable nature of the disease pose significant challenges, particularly in early identification—a formidable task confronted by the global healthcare system [2]. Regrettably, compliance with sepsis bundling remains suboptimal, and each hour of delay in completing sepsis bundling treatment amplifies the relative risk of mortality by 4% [3]. As a diagnostic and prognostic tool for sepsis, high sensitivity is imperative. The Quick Sepsis-related Organ Failure Assessment (qSOFA) score, introduced in Sepsis 3.0, serves as a valuable reference for frontline clinicians in disease diagnosis. Nevertheless, a study by Flavia R. et al. posits that qSOFA may inadequately identify a substantial cohort of high-risk patients, and clinical practitioners may exhibit a proclivity to misuse it. The inclination to employ qSOFA as an exclusionary screening tool for sepsis contradicts the Sepsis 3.0 recommendation, thereby risking delayed diagnosis and potential oversight of cases [4]. Despite ongoing updates to sepsis diagnostic criteria, the efficacy of commonly utilized diagnostic markers such as lactate and procalcitonin in diagnosis and prognosis has been scrutinized. Consequently, there exists an imperative to discern novel biomarkers to enhance diagnostic precision.

The evolution of proteomics, leveraging mass spectrometry, has yielded substantial insights into comprehending the potential molecular mechanisms underlying diseases, particularly facilitating protein component classification. Within this domain, data-independent acquisition (DIA) has garnered popularity owing to its enhanced capabilities in detection and quantification. The underlying principle involves the isolation and fragmentation of precursor ions within predefined windows, followed by the analysis of all resulting fragment ions through a high-resolution mass spectrometer. Consequently, DIA exhibits notable attributes such as heightened reproducibility, accuracy, and profound capabilities for proteomic analysis, enabling extensive screening of differentially expressed proteins (DEPs). This, in turn, renders DIA conducive to integration into large-scale biomarker research initiatives. The synergistic application of DIA with other advanced technologies holds promise for further augmenting analytical performance [5–7]. Subsequently, the enzyme-linked immunosorbent assay (ELISA) emerges as an analytical technique employed for the qualitative or quantitative analysis of target proteins. ELISA relies on the enzymatic catalysis-induced color development and color intensity of the substrate, thereby facilitating accurate assessment. This methodology proves invaluable in elucidating the presence and concentration of specific proteins, contributing to a comprehensive understanding of molecular events and paving the way for diverse applications in both basic research and clinical diagnostics.

Since its inception, molecular biology has undergone notable transformations. As research endeavors have deepened, it has become evident that explicating large and intricate systems through the study of individual components is insufficient. In response, the field of bioinformatics has emerged and found widespread application, particularly in the processing of large-scale mass spectrometry data. A prominent tool in this context is Gene Ontology (GO), a frequently employed enrichment analysis method for investigating gene or protein functions. This classification system delineates functions into biological processes, cellular components, and molecular functions. GO analysis aids researchers in comprehending the biological processes in which target genes or proteins may participate, the cellular environments they inhabit, and the molecular functions they fulfill. Another crucial resource is the Kyoto Encyclopedia of Genes and Genomes (KEGG), which facilitates the analysis of signaling pathways involved and provides insights into the specific pathways where differentially expressed genes/proteins converge. This knowledge is instrumental in understanding the broader functional context of the studied genes or proteins. In conjunction with these resources, the construction of a protein-protein interaction (PPI) network is pivotal. This involves connecting each protein as a node and establishing edges with other related proteins to form a complex network. Utilizing specialized software, the interconnections and interactions between various proteins in the network can be described, enabling the prediction of potential core biomarkers. This integrated approach, encompassing GO, KEGG, and PPI analyses, enhances our ability to unravel the intricate relationships and functionalities within molecular systems [8–10].

Biomarkers play a pivotal role in enhancing early diagnosis and the classification of disease severity, thereby facilitating targeted management and appropriate treatment strategies. This, in turn, contributes to the formulation of precise therapeutic approaches, ultimately improving the overall quality of life for patients. The integration of Data-Independent Acquisition (DIA) and enzyme-linked immunosorbent assay (ELISA) technologies enables large-scale screening for differentially expressed proteins (DEPs). Augmented by bioinformatics, this approach enhances the positivity rate of predictive biomarkers, establishing connections with the clinical information of patients. This integrated methodology expedites the identification of potential biomarkers with diagnostic and prognostic value. As a result, it introduces novel diagnostic tools into clinical practice, offering a transformative impact on the early detection and management of diseases [11].

## Materials and methods

### Subject recruitment and blood collection

The specimens utilized in this experiment were derived from peripheral blood samples obtained from sepsis patients (*n* = 56) and healthy volunteers (*n* = 13) hospitalized in the Emergency Medicine Department (EICU) of Southwest Medical University Affiliated Hospital. The sampling period encompassed January 2019 to December 2019. Inclusion criteria comprised: (1) Admission to the Emergency Department EICU with a diagnosis of sepsis; (2) Adherence to diagnostic criteria outlined in the Sepsis 3.0 guidelines jointly released by the American Society of Critical Care Medicine (SCCM) and the European Society of Critical Care Medicine (ESICM) in 2016, denoted by an infection + SOFA score of ≥ 2; (3) Age of the patient ranging from ≥ 14 years old to ≤ 70 years old; (4) Voluntary agreement by the patient or their legal representative to participate in the experiment, substantiated by the signing of an informed consent form. Exclusion criteria entailed: (1) Prior organ failure; (2) Pre-existing history of immune system diseases; (3) Pre-existing history of blood system diseases; (4) Patient unwillingness to participate in the experiment. Ethical considerations were paramount, and the experiment was conducted under the auspices of the Ethics Committee of Southwest Medical University Affiliated Hospital (Ethics Number: ky2018029). Each specimen collection was accompanied by the requisite signed approval from the patient or their family member. The clinical trial associated with this experiment is registered under the identifier ChiCTR1900021261. Concurrently, pertinent clinical test data were collated from the participating patients.

### Obtaining protein data through DIA mass spectrometry analysis

Among the acquired samples, a cohort of 22 individuals diagnosed with sepsis was randomly designated as the sepsis group, whereas a control contingent comprising 10 ostensibly healthy volunteers was identified as the normative cohort for subsequent data-independent acquisition (DIA) proteomics scrutiny. Employing the Q-Exactive HF instrument from Thermo Fisher Scientific, situated in San Jose, CA, mass spectrometry analysis of protein hydrolytic peptides within the specimens was conducted. This procedure yielded precise and iteratively reproducible full mass spectrometry data across a substantial array of proteins in DIA mode. Concomitantly, leveraging the spectrum library, which was constructed via the conventional data-dependent acquisition (DDA) model, coupled with the utilization of the MSstats software, facilitated both qualitative and quantitative analyses of the peptides and proteins discerned through mass spectrometry interrogation. The outcomes of these analyses engendered a plethora of dependable quantitative data points [12].

### Screening target proteins

All data undergo logarithmic processing. Principal Component Analysis (PCA) is employed to assess the inter-group discriminability of the collected samples. R version 4.2.1 is utilized for statistical analysis and visualization during the screening for Differentially Expressed Proteins (DEPs) within both the normal and sepsis groups. The screening criteria include a Foldchange ≥ 2 and a False Discovery Rate (FDR) < 0.05. The identified DEPs are subsequently submitted to the STRING online platform (https://string-db.org/) where an online platform has been established, and a Protein-Protein Interaction (PPI) network is constructed. The network is then visualized using Cytoscape version 3.8.0 software to pinpoint differentially expressed proteins at the core of the network between the normal and sepsis groups. Simultaneously, a search for potential serum biomarkers is conducted in the PubMed database [13].

### Functional and pathway enrichment analysis

To deepen our comprehension of the functions and roles of Differentially Expressed Proteins (DEPs) in sepsis, we conducted Gene Ontology (GO) and Kyoto Encyclopedia of Genes and Genomes (KEGG) enrichment analyses using the Xiantao Academic Platform (www.xiantao.love). This analytical approach allows us to elucidate the potential involvement of DEPs in specific biological processes or signaling pathways. Through the enrichment analysis, we aim to provide a preliminary explanation for the biological contexts or pathways in which the identified DEPs may be implicated, shedding light on their potential functional significance in the context of sepsis.

### ELISA validation

To ascertain the expression levels of Resistin, enzyme-linked immunosorbent assay (ELISA) validation was conducted on all initially collected blood samples. Regrettably, due to challenges related to specimen preservation, a subset of samples was excluded from the analysis. Consequently, the sepsis group (*n* = 53) and the normal group (*n* = 12) were the cohorts ultimately subjected to validation. In this study, the ELISA double antibody sandwich method was employed to quantify Resistin levels in the samples. The procedure involved introducing the specimen to a microtiter plate coated with Resistin antibodies, followed by binding with horseradish peroxidase (HRP)-labeled detection antibodies, forming an antibody-antigen-enzyme-labeled antibody complex. Subsequent steps included washing, the addition of TMB color reagent for chromogenic development, and measurement of absorbance at a wavelength of 450 nm using an enzyme-linked immunosorbent assay (ELISA). The content of Resistin in the sample was then calculated based on the standard curve. It is noteworthy that the color intensity of the sample is directly proportional to the content of Resistin, providing a quantitative measure of the target protein in the specimens [9].

### Analysis of subject work characteristic curve

Leveraging Hiplot Pro (https://hiplot.com.cn/), a comprehensive web service tailored for biomedical data analysis and visualization, we employed ROC analysis tools to scrutinize and visualize Receiver Operating Characteristic (ROC) curves. Specifically, the ROC curve was drawn to assess the diagnostic capability of Resistin, and the area under the curve (AUC) was calculated. The AUC of the ROC curve, which compares the normal group and the sepsis group, surpassed 0.7. This observation led us to assert that Resistin exhibits diagnostic efficacy in distinguishing between the normal and sepsis groups. The AUC value serves as a quantitative metric for evaluating the discriminatory power of Resistin as a potential diagnostic biomarker in the context of sepsis.

### Statistical analysis

The statistical analysis of ELISA data and patient clinical information was conducted using SPSS 22.0 software and GraphPad Prism software 9.0 (GraphPad Inc., La Jolla, CA). Continuous variables in clinical data were expressed as median (interquartile range), and the Mann-Whitney U-test was employed for non-parametric tests. Routine measurement data were presented as mean ± standard deviation, and statistical analysis was performed using the non-paired t-test. Categorical variables were analyzed using the chi-square test, and Fisher’s exact test was employed for the comparison of the number of patients. The significance level was set at a cut-off value of *P* < 0.05. This comprehensive analytical approach ensures robust statistical assessment and graphical representation of both ELISA data and clinical variables, enhancing the reliability and interpretability of the study findings.

## Result

### Clinical characteristics of the experimental subjects

The clinical data of the patients enrolled in this study are summarized in Table 1. The study cohort comprised 10 normal individuals and 22 patients diagnosed with sepsis. There were no statistically significant differences in terms of gender and age between the sepsis patients and the normal volunteers. However, notable differences (*P* < 0.05) were observed in white blood cell (WBC) count, neutrophil count, and alanine aminotransferase levels between the normal control group and sepsis patients. Conversely, no significant difference was found in creatinine levels. Furthermore, a significant increase in procalcitonin (PCT) and lactate (Lac) levels was observed in the sepsis group compared to the normal volunteers. These findings highlight key distinctions in various clinical parameters between the normal and sepsis cohorts, providing valuable insights into the clinical characteristics of the study participants.

**Table 1:** The clinical data of the patients enrolled in this study

Clinical variables	Normal Control(*n* = 10)	Sepsis(*n* = 22)
Male	5(50%)	14(64%)
Female	5(50%)	8(36%)
Age(year)	53.5 ± 7.66	35.5 ± 4.95
WBC(10^9^/L)	6.88 ± 1.85	13.55 ± 6.91^*^
NEUT(10^9^/L)	4.13 ± 1.09	14.31 ± 12.98^*^
ALT(u/L)	20.90(16.13, 26.50)	34.35(20.4, 58.43)^*^
Cre(umol/L)	64.40(53.03, 72.58)	66.00(44.62, 102.28)
PCT(ng/mL)	NA	4.46(1.48, 37.77)
Lac(mmol/L)	NA	2.87(2.18, 4.41)

### Identification of differential proteins in sepsis

**Fig. 1:**
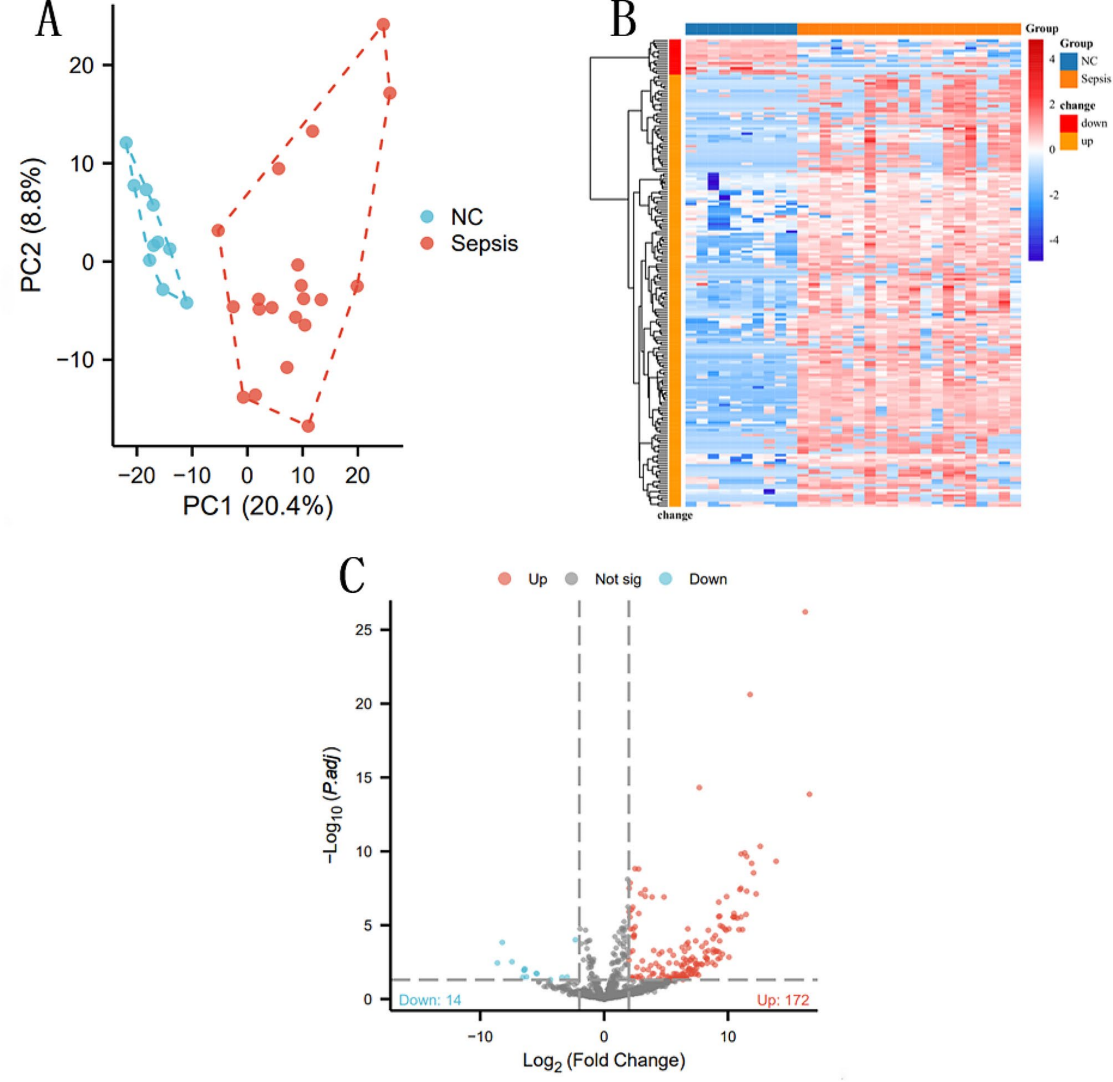
**A**: Sepsis sequencing data quality control. PCA analysis showed that the samples could be distinguished by the main genes, and there were no outliers. **B**,**C**: Differential screening of sepsis sequencing data. Compared with the normal group, 186 differential genes were screened in the samples of the sepsis group, and 14 were down-regulated and 174 were up-regulated; Compared with the normal group, red indicates an upward trend, while blue indicates a downward trend

Principal Component Analysis (PCA) revealed a notable discriminability between normal and sepsis samples in the Data-Independent Acquisition (DIA) based proteomics analysis (Fig. 1A). No outliers were identified during this analysis. The proteomics data were rigorously examined using R version 4.2.1. Subsequently, 187 Differentially Expressed Proteins (DEPs) were identified through screening serum samples from patients in the normal and sepsis groups. In comparison to the normal group, the sepsis group exhibited 14 downregulated protein expressions and 172 upregulated gene expressions (Fig. 1B C). These findings underscore the molecular distinctions between the normal and sepsis groups, shedding light on specific proteins and genes that may be implicated in the pathophysiology of sepsis.

### Protein interaction networks and core protein analysis

**Fig. 2:**
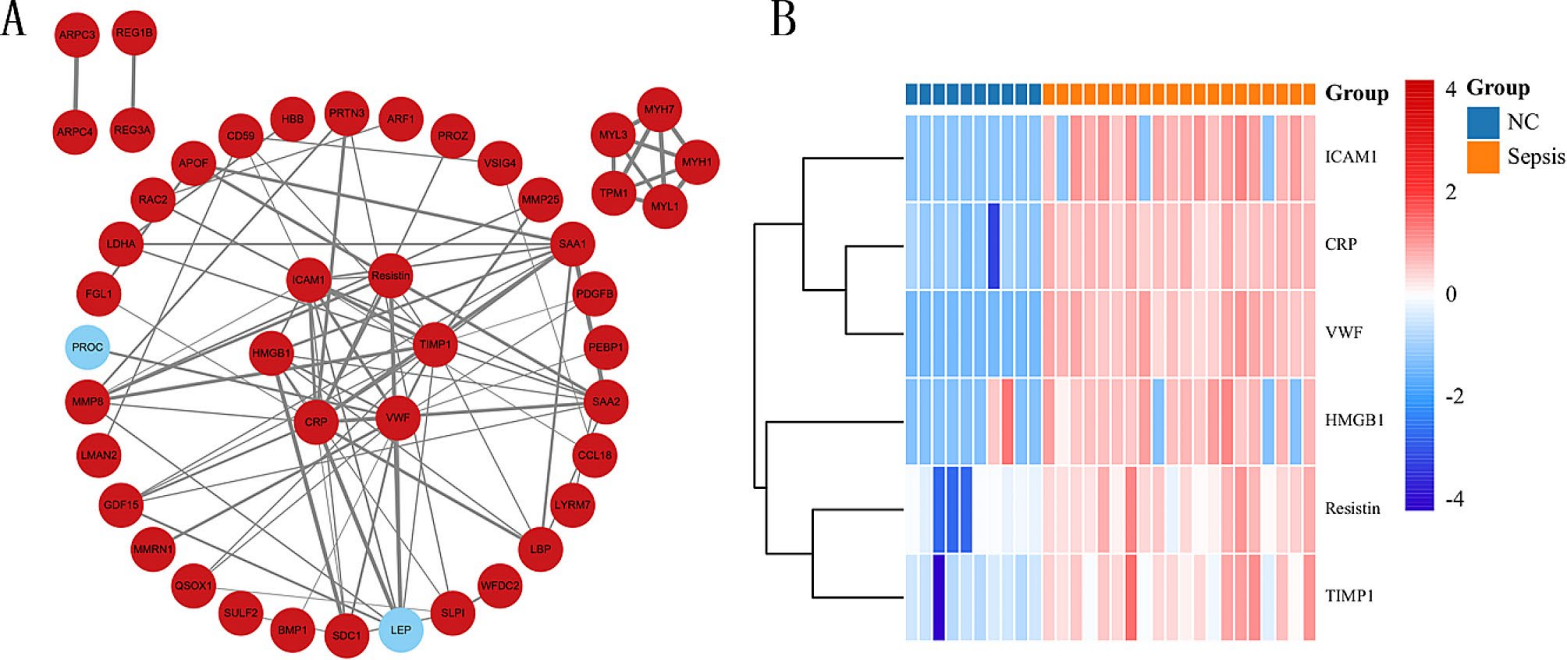
Protein-protein-interaction network. **A**, **B**: ICAM1, Resistin, TIMP1, VWF, CRP, and HMGB1 Identified as core genes, it exhibits upregulation in expression compared to the normal group

The Differentially Expressed Proteins (DEPs) identified were submitted to the STRING online platform (https://stringdb.org) to construct a Protein-Protein Interaction (PPI) network (Fig. 2A). This network visualizes the relationships between proteins. Notably, within the PPI network analysis, key proteins including ICAM1, Resistin, TIMP1, VWF, CRP, and HMGB1 were identified at the central nodes of the network module, revealing a significant difference (*P* < 0.05) between the normal group and the sepsis group (Fig. 2B). A literature search in the PubMed database did not yield relevant studies on the levels of Resistin in the serum of sepsis patients. Consequently, the specific role of Resistin in sepsis warrants further validation and investigation. These findings contribute to a comprehensive understanding of the interplay between proteins in the context of sepsis and highlight potential candidates for further exploration.

### DEPs for GO and KEGG enrichment analysis

**Fig. 3:**
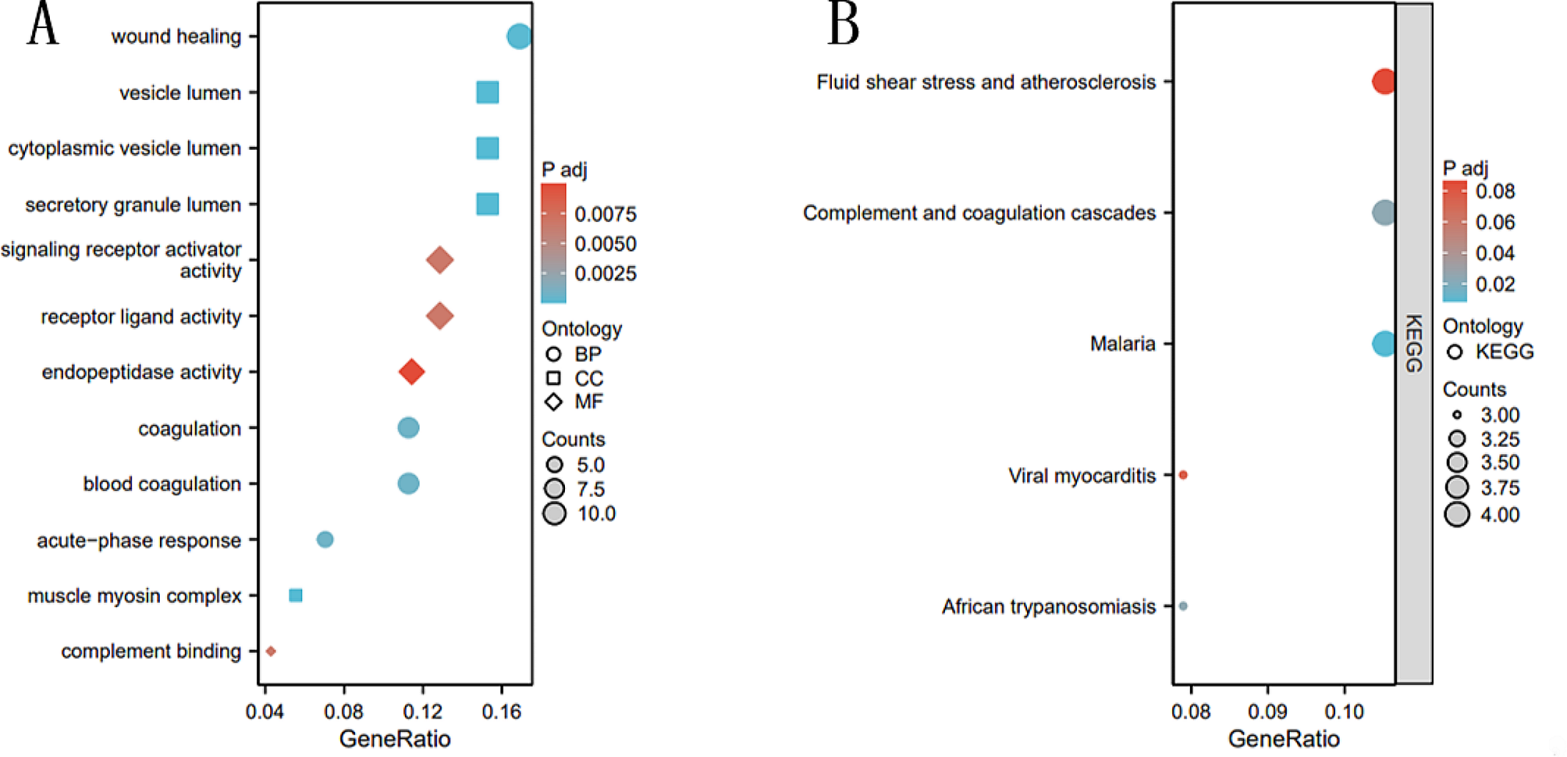
**A**. GO analysis bubble plot of candidate differential genes. Different shapes represent different functions, dot size represents the number of enriched genes, and color represents enrichment significance. **B**. KEGG analysis bubble plot of candidate differential genes. Different shapes represent different functions, dot size represents the number of enriched genes, and color represents enrichment significance

The Gene Ontology (GO) analysis, employed for functional enrichment of Differentially Expressed Proteins (DEPs), revealed their predominant involvement in various biological processes, including wound healing, coagulation, and blood coagulation. Additionally, these proteins were found to be associated with specific cellular components such as vesicular cavities, cytoplasmic vesicular cavities, and secretory granule cavities. The molecular functions of these DEPs encompassed signal receptor activation activity, receptor ligand activity, and endopeptidase activity (Fig. 3A). Furthermore, Kyoto Encyclopedia of Genes and Genomes (KEGG) pathway analysis indicated that the DEPs are primarily associated with complement pathways and signaling pathways related to conditions such as malaria (Fig. 3B). These findings provide valuable insights into the diverse functional roles and potential pathways through which the identified proteins may contribute to the pathophysiology of sepsis.

### Verify Resistin through ELISA

**Fig. 4:**
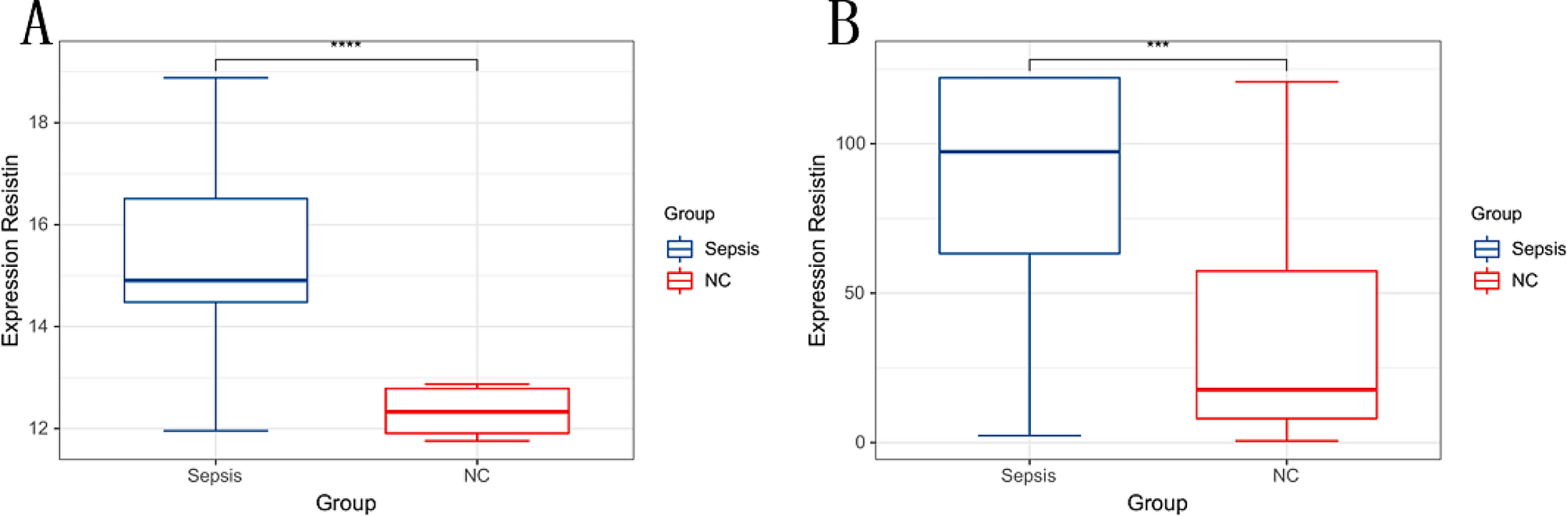
**A**. Comparison of resistin expression between sepsis and normal groups in randomly selected samples. The sepsis group was significantly higher than the normal group. **B**. The sepsis group compared to the normal group in all samples. The result is still significantly high expression in the sepsis group

According to the results presented in Fig. 4A, the expression level of Resistin in the serum of randomly selected sepsis patients was measured at 15.3 ± 1.8 ng/mL, significantly surpassing that of the normal control group (12.3 ± 0.4 ng/mL) (*P* < 0.01) (Fig. 4A). Furthermore, in the overall sample, the expression level of Resistin in the sepsis group (86.1 ± 36.5 ng/mL) remained significantly higher than that of the normal control group (32.8 ± 35.2 ng/mL) (*P* < 0.01) (Fig. 4B). These observations strongly suggest a positive correlation between the elevated expression level of Resistin and the occurrence of sepsis.

### ROC curve analysis diagnostic performance

**Fig. 5:**
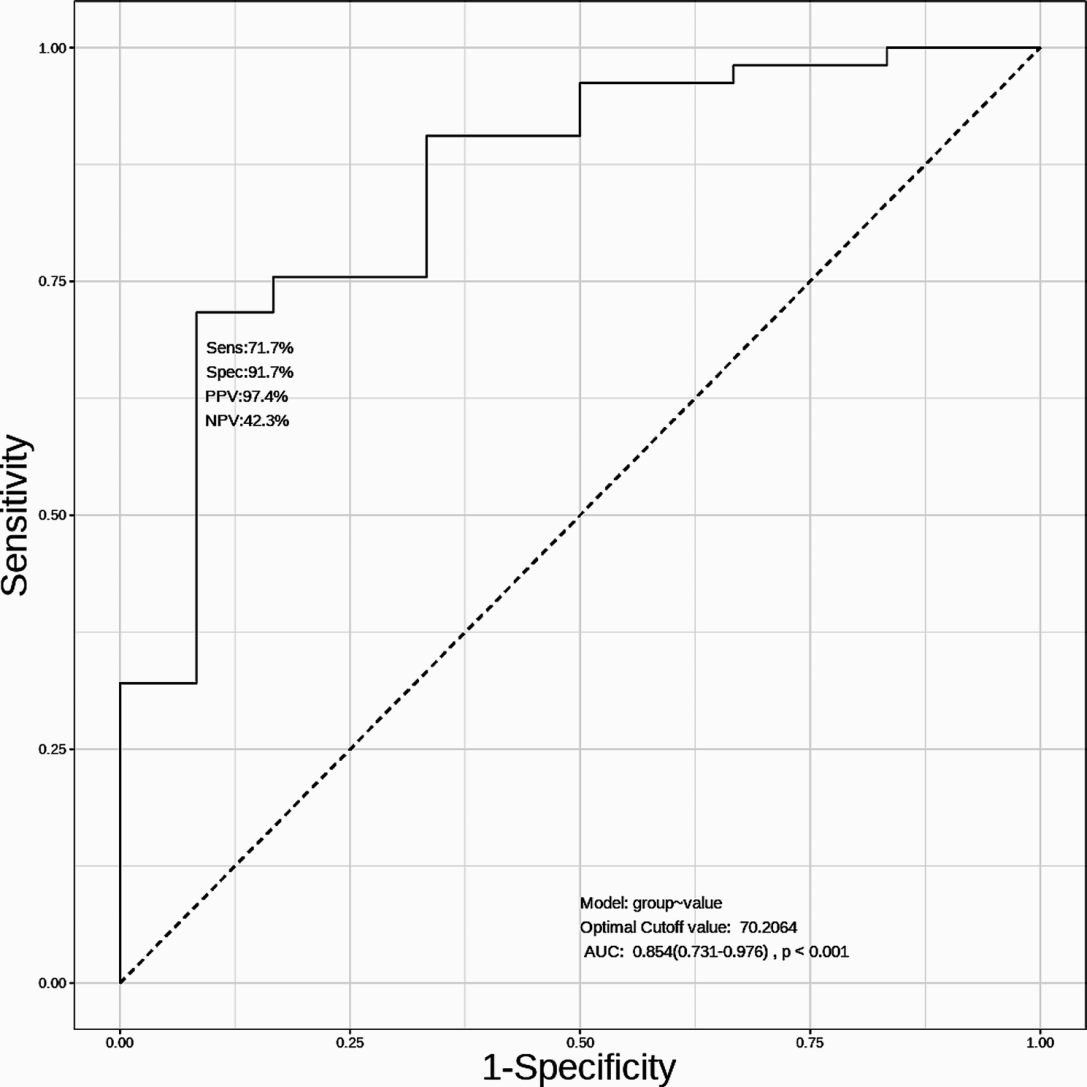
ROC curves of Resistin. AUC: 0.854, sensitivity:71.7%, specificity: 91.7%

ROC curves were generated based on the ELISA results, and the analysis revealed that Resistin exhibits substantial diagnostic value for sepsis. The area under the ROC curve was calculated to be 0.854, reflecting a robust discriminatory performance. The sensitivity of Resistin as a diagnostic marker for sepsis was determined to be 71.7%, while the specificity reached 91.7% (Fig. 5). These findings underscore the potential of Resistin as a valuable biomarker for distinguishing sepsis cases, demonstrating both sensitivity and specificity in diagnostic applications.

## Discussion

Sepsis represents a formidable threat to global health, characterized by symptoms such as heightened body temperature, accelerated heart rate, and shallow breathing upon disease onset. As the condition progresses, it may advance to shock or systemic multi-organ dysfunction. Early detection and proactive intervention play pivotal roles in reducing mortality and disability rates associated with sepsis. This study employed bioinformatics and Data-Independent Acquisition (DIA) analysis to meticulously screen and identify Resistin as a potential biomarker for sepsis. Functional enrichment analysis revealed its involvement in various biological processes and pathways associated with infection response, immunity and leukocyte activation or degranulation. Consistent with the bioinformatics predictions, our ELISA results substantiate a significant elevation in Resistin expression among individuals affected by sepsis. Since the small discovery set for LC-MS was also the samples for ELISA that the results represent a technical proof of principle. The Receiver Operating Characteristic (ROC) curve analysis further underscores the diagnostic prowess of Resistin in identifying sepsis cases. The recognition of Resistin as a potential biomarker holds promise for enhancing early detection and targeted therapeutic interventions in the context of sepsis management.

Resistin, situated on chromosome 19p13.2, is a cysteine-rich secreted protein encoded by the RETN gene, primarily expressed in white blood cells. Its structural configuration comprises a carboxyl-terminal disulfide on a positively charged surface β Sandwich “head domain,” and an amino-terminal with negative electrostatic potential α Spiral tail chain [14]. Although associated with inflammatory responses, its precise role in host defense and signal transduction mechanisms remains elusive. Notably, studies suggest a potential link between Resistin and the accelerated accumulation of low-density lipoprotein in arteries, a factor closely tied to cardiovascular and cerebrovascular diseases in various metabolic, chronic inflammatory, or malignant conditions, where serum Resistin levels aberrantly increase [14, 15]. Propheter and Harris et al.‘s work, employing transmission electron microscopy, demonstrated Resistin as a membrane-permeabilizing protein, forming polymeric pores by binding to bacterial lipids, indirectly highlighting its antibacterial potential and ability to induce bacterial cytoplasmic leakage [16]. Tarkowski et al.‘s findings further revealed Resistin’s capacity to stimulate the release of pro-inflammatory cytokines, such as IL-1, IL-6, and TNF-α, from human leukocytes, monocyte line THP-1, and epithelial cells (HEK6), activating downstream NF-κB and MAP kinase signaling pathways through the TLR4 pathway [17]. However, Miller et al.‘s study posits that Resistin may disrupt immune responses, impairing neutrophil-mediated microbial killing and potentially inducing immunosuppression [18]. This dichotomy positions Resistin as a dual-function molecule, acting as both a pro-inflammatory factor, mediating cytokine storms, and exhibiting anti-inflammatory activity, implicating it in the complex immunosuppressive processes central to sepsis development. A comprehensive understanding of Resistin’s role in sepsis could potentially unveil novel targets for therapeutic interventions.

As with previous studies, this research aims to identify biomarkers with diagnostic or therapeutic potential for sepsis using DIA and ELISA methods. However, the single-center nature of the study limits the generalizability of the conclusions. Unlike earlier studies, which predominantly sourced samples from Europe and America, this research addresses the lack of data on East Asian populations, particularly those represented by Chinese individuals. Additionally, some previous studies collected samples prior to the publication of Sepsis 3.0 guidelines, limiting the relevance of their conclusions to the current sepsis landscape. Previous methodologies often relied on clinical data collection, which is not conducive to subsequent functional and mechanistic studies. In contrast, this study employs proteomic analysis rather than transcriptomic approaches, directly revealing protein expression, structure, and functional changes. This provides direct evidence for understanding life processes and disease mechanisms, as the occurrence and progression of many diseases are closely linked to abnormal protein expression and functional alterations [19–22]. 

This study integrates proteomics and bioinformatics. Proteomics technology enables an in-depth investigation into the function and mechanisms of proteins, while bioinformatics excels in efficiently processing and interpreting large-scale biological data. The combined application of these disciplines allows for a comprehensive elucidation of the molecular structure and function of living organisms. This approach facilitates a deeper understanding of physiological and pathological changes, enabling precise exploration of key proteins and their interactions related to disease occurrence, progression, and treatment. In this study, the differential protein Resistin was identified using DIA and ELISA techniques. Functional enrichment analysis revealed its involvement in various biological processes and pathways. The potential role of Resistin in sepsis was preliminarily elucidated, and the reliability of these findings was confirmed through ELISA validation. This provides a foundation for subsequent mechanistic studies.

There are many examples of resistin measured in blood cells, but very few in serum. And there are few publications showing the detection of resistin through mass spectrometry, which have been confirmed through ELISA.But we still have to admit some limitations of the current research. Firstly, the study is centered on sepsis patients, and to establish Resistin as a viable biomarker for sepsis, comparisons with individuals not afflicted by sepsis are imperative. Secondly, the sample size in this study is relatively small, and gender stratification was not possible. Future research endeavors should focus on expanding the sample size and conducting multicenter studies to enhance the generalizability of the findings. Lastly, the specific mechanistic actions of Resistin in sepsis remain unclear, necessitating further experimental investigations to validate and elucidate the observed associations. Addressing these limitations will contribute to the robustness and comprehensiveness of the study’s findings and their potential implications in clinical settings.

## Conclusion

In conclusion, the findings from Data-Independent Acquisition (DIA) and Enzyme-Linked Immunosorbent Assay (ELISA) analyses collectively indicate a significant elevation of Resistin in the serum of sepsis patients. The observed differential expression of Resistin holds notable significance in the context of sepsis, suggesting its potential utility as a diagnostic marker for this condition. These results underscore the promising application value of Resistin as a candidate biomarker for sepsis diagnosis. Further research, including comparisons with non-sepsis cohorts, larger sample sizes, and elucidation of Resistin’s mechanistic role, is warranted to validate and expand upon these findings.

## Data Availability

No datasets were generated or analysed during the current study.
